# Implementation of evidence on the nurse-patient relationship in psychiatric wards through a mixed method design: study protocol

**DOI:** 10.1186/s12912-016-0197-8

**Published:** 2017-01-11

**Authors:** Antonio R. Moreno-Poyato, Pilar Delgado-Hito, Raquel Suárez-Pérez, Juan M. Leyva-Moral, Rosa Aceña-Domínguez, Regina Carreras-Salvador, Juan F. Roldán-Merino, Teresa Lluch-Canut, Pilar Montesó-Curto

**Affiliations:** 1 Escola Superior d’Infermeria del Mar, Parc de Salut Mar (Pompeu Fabra University associated center), Aiguader, 80, 08860 Barcelona, Spain; 2 School of Nursing, University of Barcelona, Feixa Llarga s/n. 08907 L’Hospitalet del Llobregat, Barcelona, Spain; 3 Institut de Neuropsiquiatria i Addiccions, Parc de Salut Mar, Pg. Marítim, s/n. 08860, Barcelona, Spain; 4 School of Nursing, Autonomous University of Barcelona, Avda. Can Domenech, Edifici M. 08193 Bellaterra (Cerdanyola del Vallès), Barcelona, Spain; 5 Campus Docent Fundació Privada Sant Joan de Déu. School of Nursing, University of Barcelona, Santa Rosa, 39-57, 08950 Esplugues de Llobregat, Spain; 6 School of Nursing, Rovira i Virgili University, Avda Remolins 13-15, Tortosa, 43500 Tarragona Spain

**Keywords:** Psychiatric nursing, Nursing care, Therapeutic Relationship, Mixed method design, Quality of care, Participatory action research

## Abstract

**Background:**

Psychiatric nurses are aware of the importance of the therapeutic relationship in psychiatric units. Nevertheless, a review of the scientific evidence indicates that theoretical knowledge alone is insufficient to establish an adequate therapeutic alliance. Therefore, strategies are required to promote changes to enhance the establishment of the working relationship. The aims of the study are to generate changes in how nurses establish the therapeutic relationship in acute psychiatric units, based on participative action research and to evaluate the effectiveness of the implementation of evidence through this method.

**Methods/Design:**

The study will use a mixed method design. Qualitative methodology, through participative action research, will be employed to implement scientific evidence on the therapeutic relationship. A quasi-experimental, one-group, pre-test/post-test design will also be used to quantitatively measure the effectiveness of the implementation of the evidence. Participants will consist of nurses and patients from two psychiatric units in Barcelona. Nurses will be selected by theoretical sampling, and patients assigned to each nurses will be selected by consecutive sampling. Qualitative data will be gathered through discussion groups and field diaries. Quantitative data will be collected through the Working Alliance Inventory and the Interpersonal Reactivity Index. Qualitative data will be analysed through the technique of content analysis and quantitative data through descriptive and inferential statistics.

**Discussion:**

This study will help to understand the process of change in a nursing team working in an inpatient psychiatric ward and will allow nurses to generate knowledge, identify difficulties, and establish strategies to implement change, as well as to assess whether the quality of the care they provide shows a qualitative improvement.

## Background

The Therapeutic Relationship (TR) is one of the most important tools at nurses’ disposal, especially in mental health nursing. Indeed, the concept of the therapeutic relationship emerged in parallel to the professionalization of nursing care [1] and is considered the cornerstone of psychiatric and mental health nursing [2]. Adequate establishment of the therapeutic relationship increases the efficacy of any nursing intervention in the acute mental health setting [3].

Multiple terms are used to describe the same concept. The TR is also called the helping relationship, the nurse-patient relationship, the trusting relationship, and the therapeutic alliance. Nevertheless, the central focus of all these concepts is the helping working relationship [4]. Likewise, the concept of the TR has been enriched by diverse perspectives and paradigms, including the psychodynamic perspective originating with Freud, the pantheoretical model, and the humanist or person-centred approach. Consequently, some authors have stated that personal qualities rather than theoretical orientation take centre stage in the TR [4].

Psychiatric nurses are aware of the concept of the TR and its importance [2, 4–10]. However, they are also aware that specific skills are required to develop and maintain a TR with patients [7], which involves a huge effort and, moreover, implies difficulty in its achievement. Factors such as consistency, empathy, the ability to listen, making a positive first impression, a safe and comfortable environment, and teamwork are basic pillars, encouraging and aiding the development of a TR [10].

For their part, patients perceive attitudes, values and a trusting relationship as being more important than technical skills in the therapeutic relationship [4]. Service users expect to receive individual attention as part of their treatment plan [11]. Having a feeling of control and of self-determination is highly important, and this sense of meaning and control is provided by interpersonal relationships [12]. So much so, that what patients want most are empathetic nurses, ie, those able to identify what the patient expects or needs from the nurse at any given moment [4]. Patients value nurses who are patient and imaginative and have a sense of humour [9], who listen and are empathetic [4]. The greater the chronicity, the more patients lose interest in their physical needs and the more they value relational aspects of care [13].

Despite evidence of the greater effectiveness of the TR and psychodynamic training in clinical practice [14], mental health nursing has been strongly influenced by the biomedical model and has become increasingly depersonalized [15]. There is no recognition of the need for the care of emotions, or of the importance of the emotional work required to develop and maintain a quality TR [13]. In this regard, routine tasks and administrative duties, as well as the time they consume, hamper the provision of individual attention and consequently the effectiveness of the TR [11]. Nevertheless, some evidence suggests that staff spend increasingly less time with patients, despite an increase in the staff-to-patient ratio in some units [16].

For some patients, inaccessibility and lack of communication-and therefore a lack of information provided by some staff-are factors that limit the TR [17]. Likewise, lack of availability, inequality, and differences in values and experience are factors that distance nurses from patients and hamper the development and maintenance of the therapeutic alliance. Often, patients feel they are given little opportunity to collaborate in their care [17], perceive that they spend most of their time alone and that they have little relationship with the care team [16]. Some patients feel like prisoners and report that staff who are themselves insecure adopt intimidating and condescending attitudes [18].

According to nurses, the elements that most frequently hamper the therapeutic relationship are the amount of time spent on administrative duties, patients’ negative attitudes and unrealistic expectations, nurses’ inability to confront these issues with patients, and feeling inadequately trained to provide the individual attention required by patients [11]. Also important are nurses’ perception that socially unacceptable factors such as segregation, coercion and social control are working norms and that this situation is, moreover, experienced as demotivating and routine, as if working life were dominated by functions of social control [19].

In this regard, and although nurses are aware of the importance of the TR and how to foster such an alliance, the scientific evidence shows that theoretical knowledge of communicative and interpersonal skills alone is insufficient to develop these skills effectively, and hence to establish an effective therapeutic relationship and provide high-quality nursing care [3, 9, 15–17, 20–23].

Therefore, if knowledge of these skills does not necessarily lead to their use in daily clinical practice, it seems necessary to devise strategies to promote these competencies. Effecting change *for* action and *through* action is characterised by participation and reflection, which encourages both learning through action and about the action taking place.

### Theoretical framework

Participatory Action Research (PAR) is a method that encourages participatory and reflective change at the same time as it increases an understanding and transformation of practices [24]. Thus, PAR aims to achieve reliable, effective and efficient results to improve group or collective situations. PAR is a research method because it aims to increase knowledge and understanding of phenomena through exploratory scientific techniques. Moreover, PAR is also an action because it aims to intervene in the phenomenon being explored by effecting change in that phenomenon. Finally, PAR is participatory since it encourages collaboration and cooperation among all the persons involved throughout the process.

**Fig. 1:**
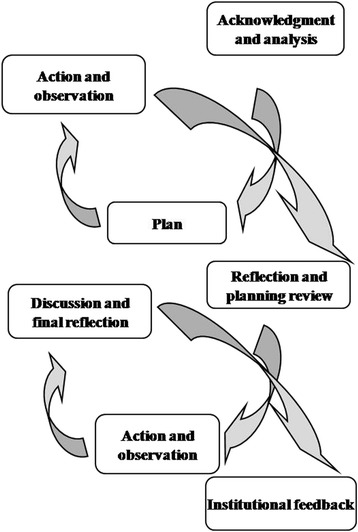
Stages of Participatory Action Research

Therefore, PAR is a dynamic method consisting of an open, holistic and egalitarian process among researchers and participants [25]; the action needs to be filtered through experience and reflection before it can improve or change practices. In PAR, knowledge generation is viewed as a collaborative process, in which the skills and experiences of each participant are essential to the outcome of the project. The goal of PAR is to resolve or modify specific problems in communities, in this case, in acute care nursing teams. The process is effected through a series of steps included in a spiral of self-reflective circles (Fig. 1): 1. Planning a change, 2. Acting and observing the process of change, 3. Reflecting on the process and its consequences, 4. Replanning, 5. Acting and observing again, 6. Reflecting again … and so on [24].

### Objectives

General:

1. To generate change in how nurses establish the therapeutic relationship in an acute psychiatric unit, based on PAR.
2. To evaluate the effectiveness of the implementation of scientific evidence through PAR, in terms of improving the therapeutic relationship, as perceived by nurses and patients, and enhancing empathy, as perceived by nurses.

Specific:

- To describe how nurses in an acute psychiatric unit perceive the establishment of the therapeutic relationship with patients.
- To identify nursing professionals’ difficulties and limitations when establishing a therapeutic relationship with a patient.
- To identify the elements of change and improvement in the establishment of the therapeutic relationship, based on contrasting the evidence with real-world clinical practice.
- To qualitatively evaluate the effects of the implementation of evidence on the development of the therapeutic relationship between nurses and patients.
- To analyse the impact of the implementation of scientific evidence on the establishment of the therapeutic relationship on:
- Measurement of the therapeutic relationship, evaluated by staff before and after the PAR process.
- Measurement of the therapeutic relationship, evaluated by patients before and after the PAR process.
- Measurement of empathy, evaluated by staff before and after the PAR process.

## Methods/Design

### Study design

**Fig 2:**
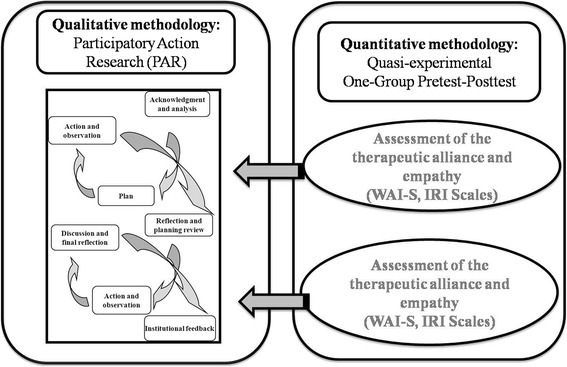
Study design

An overall design for the research project is proposed, following a mixed sequential transformational method [26]. The qualitative method will guide the process and will be the central axis of the design. Nevertheless, the sequential use of quantitative methods during the study will serve to control the process [24] and will complement analysis of the results (Fig. 2).

Therefore, to achieve the first objective, a qualitative design will be used in the form of PAR, within the constructivist paradigm and following the model of Kemmis and McTaggart [24] in cycles and stages. PAR is a method that integrates research and social action [25] and, during its implementation, includes the following stages: a) analysis of the situation, b) identification of problems and design of strategies for the proposed action, c) implementation of the strategies designed, and d) observation and systematic reflection on their implementation.

To achieve the second objective, a quasi-experimental study of a single pre-test/post-test group is proposed, with evaluation of the implementation of the evidence through PAR [27].

### Setting and participants

The study will be conducted in the acute psychiatric admissions ward of the Institute of Neuropsychiatry and Addictions (*Institut de Neuropsiquiatria i Addiccions* [INAD]) of Parc de Salut Mar de Barcelona (Spain): the Institute consists of 4 units with 107 in-patient beds for patients with acute psychiatric disorders. These units cover a population of approximately 650,000 inhabitants in the City of Barcelona, Badalona and Santa Coloma de Gramanet.

Study participants will consists of nurses working in the acute-stay units and patients admitted to these units. Theoretical sampling will be carried out in nurses. The sample will be composed of 9–10 participants representing the distinct socio-occupational profiles in the units. Thus, the group will be composed of nurses from each of the three shifts, by specialist and generalist nurses, and by staff and supply nurses. The aim is to guarantee the principles of heterogeneity and accessibility [28].

We estimate that about 30 patients will be allocated to each participating nurse in the phase prior to the start of the groups, and consequently, accepting an alpha risk of 0.05 and a beta risk of 0.2 in the bilateral contrast, nine participants will be needed to detect a difference equal to or higher than 10 units in the Working Alliance Inventory-Short (WAI-S). A standard deviation of 10 will be accepted, and a loss to follow-up rate of 10%. Consecutive sampling will be performed. Inclusion criteria will consist of the following: a) Being assigned to a participating nurse, b) Having established a link with the participating nurse during a hospital stay longer than 3 days, b) voluntarily accepting to participate in the project and providing signed informed consent.

Exclusion criteria will consist of a) showing signs of the initial stages of psychomotor agitation, and b) being subjected to physical restraint, either currently or within the last 24 h.

### Qualitative data collection

Qualitative data will be generated through discussion groups and field diaries kept by participating nurses and researchers.

### Discussion groups

Due to the importance of team work and the consequent collective social discourse distinct from individual discourse [29], it is useful for nurses to debate the objectives of the research as a group. Four group sessions will be held to cover the distinct PAR stages.

### Reflective diary

Participating nurses will construct a narrative of their own observations of the establishment of the therapeutic relationship with patients and will conduct a reflective analysis of these observations [30]. To help aid self-observation and subsequent reflection, the nurses will be provided with a dossier-guideline specifying how to conduct the self-observation and subsequent reflection.

The researcher’s field diary will help to monitor the research process [31], both descriptively and methodologically, and will help to integrate theory and practice.

### Quantitative variables and data collection

The following variables and measurement instruments will be used for quantitative data.

Variables:

1. Sociodemographic and occupational variables in nurses, and sociodemographic and clinical variables in patients.
2. Dependent variables
  The nurse-patient relationship, from the nurse’s perspective, evaluated through the WAI-S scale, therapist version [32].
  The nurse-patient relationship, from the patient’s perspective, evaluated through the WAI-S scale, client version [32].
  Nurse empathy, from the nurse’s perspective, measured with the Interpersonal Reactivity Index [33].
3. Independent variables:
  Implementation of evidence through PAR.

Instruments:

1. The Working Alliance Inventory (WAI short). This inventory measures the working alliance and therefore the nurse-patient relationship (Horvath & Greenberg 1989). The short version of this scale contains 12 items, and each item is evaluated by the health professional and patient through a scale ranging from 1 (never) to 7 (always). The higher the score, the greater the therapeutic alliance. The Spanish version of the WAI-S has good reliability and validity, with a Cronhbach’s alpha of .91 [34].
2. The interpersonal reactivity Index (IRI). The most important element in establishing the therapeutic relationship is possibly empathy. For this reason, empathy will be specifically measured through the instrument designed by Davis [33]. This index has 28 items in a Likert scale (1 = not good at all and 5 = very good). Crohnbach’s alpha for the Spanish version ranges from .73 to .71 among the 4 factors composing the IRI [34].

### Data analysis

#### Qualitative data analysis

The content analysis method will be used, which is an appropriate approach to the analysis and categorization of qualitative data according to empirical and theoretical criteria [35]. Data from the discussion groups and diaries will be transcribed literally. After verification of the authenticity of the transcriptions, the text will be fragmented in descriptive codes assigned on the basis of its content. In a second stage of the analysis, these codes will be grouped into interpretative categories, bearing in mind their similarities and differences and relating them to the main themes identified in the theoretical framework and the study objectives. This process is not linear or sequential but is rather constant, cyclic, recursive and complex, demanding the construction of data for their subsequent reconstruction and interpretation of their meaning [35, 36]. The NVivo platform, version, 10 will be used in the analysis.

#### Quantitative data analysis

The analysis will focus on numerical differences, obtained in the WAI-S and IRI, observed before and after the group phases of the PAR. Descriptive statistics will be used for sample description and evaluation of the scores. Differences between baseline scores and the post-group assessment will be estimated through the application of a parametric test (Student’s *t*-test for paired data) in the case of quantitative variables after confirmation of the normality and homogeneity of their variance; for data that do not follow a normal distribution), non-parametric tests will be used: the Wilcoxon test will be used for quantitative variables and the chi-square test or Fisher’s exact test for qualitative variables. To interpret the results, statistical significance will be set at *p* < 0.05 for a 95% confidence interval (95% CI) in the case of a bidirectional hypothesis. The analysis will be performed with the IBM SPSS Statistics 21 package.

### Ethical considerations and measures to preserve participants’ confidentiality and anonymity

The project has been approved by the Clinical Research Ethics Committee. Moreover, in compliance with Spanish Law 15/1999 of 13th December on the Protection of Personal Data, authorization for dissemination of the results of the project will be sought from all participants, guaranteeing their anonymity and strict confidentiality. All participants’ names will be codified and assigned a number. The data obtained will be added to an archive named “MERTCEATE DATA” and the information provided by the questionnaires will be completely confidential. Any data or names that could be used to identify the participants will be eliminated. The data will be stored for the purposes of this research project. Participation among patients and health professionals will be voluntary and all participants will be required to provide their written informed consent indicating their acceptance of the study conditions and will retain the right to withdraw from the study at any point.

### Rigor

The criteria of trustworthiness and authenticity will be taken into account [37]. Trustworthiness will be provided by elements such as the study duration and the use of triangulation of techniques and researchers, while the transferibility of the results will be facilitated by the diversity of the sampling and description of the context. Compliance with the criteria of confirmability will be ensured by audio recording of discussion groups. Authenticity will be provided by the dynamics of the process, which will involve constant interaction between the realities of the observers and the realities observed. Moreover, the central quality criteria, and that forming the basis of the project, will be reflectiveness, understood as a process of returning to allow critical contrasts to be made of the processes occurring during the development of the research [38].

## Discussion

This study will help to understand the process of change in a nursing team in an inpatient psychiatric unit. The aim is that the nurses themselves will evaluate and reflect on their own interventions so that they will subsequently be able to use the scientific evidence provided by the study to assess whether the quality of the care they provide shows a qualitative improvement. To do this, we hope to learn how participants perceive the establishment of the nurse-patient relationship. As previously mentioned, the concept of the therapeutic relationship can by understood very differently by the distinct actors providing healthcare in acute-stay units and its conceptualization and contextualization is advisable as a prior step to understanding the scientific evidence. Subsequently, difficulties in implementing the evidence, both in nurses individually and in the rest of the team, will allow the planning of strategies for change. Lastly, the study aims to identify the effects of the interventions proposed and conducted by the nurses themselves so that, through a final group reflective session, a definitive report can be drawn up on proposed improvements. The use of validated measurement instruments will help to analyse, contrast, and demonstrate the change produced.

### Limitations

The duration and intensity of the research is a factor to be considered, as some participants may be lost. Therefore, it was decided to increase the size of the group sample. In addition, difficulties may appear that are specific to discussion groups, such as differences among the functioning of the groups, or limitations related to the reflective diaries, such as irregular entries or subjective interpretations by participations of situations that are foreign to their experience. The strategies that will be used to minimize difficulties and limitations are as follows: the use of known but neutral spaces to hold the groups, avoidance of simultaneous note-taking, placement of the recording device in a barely-visible area, and a priori determination of the maximum duration of the group session. The group interviewer and facilitator will be an expert in individual and group interviews and trained in communication skills. Participants will also be given documented tools to help them keep a reflective diary. Other limitations concern those inherent to a quasi-experimental design, such as the lack of a control group and the non-randomization of the sample.

## Conclusions

The therapeutic relationship is an essential component in psychiatric and mental health nursing. Improving its establishment through participative practice by clinical nurses will decrease the gap between the scientific evidence and its practical application, so that:

1. The same nurses that generate knowledge of the therapeutic relationship will establish the recommendations and strategies for its application and will identify deficiencies that form the basis for future training.
2. Nurses will be able to participate in adapting the strategies to implement changes and may even anticipate demands; this democratic and participative process will contribute to the efficiency of the intervention.
3. Facilitate the identification of difficulties in the therapeutic relationship and areas requiring exploration, which may open new lines of research.
4. Improvement in the therapeutic relationship in psychiatric inpatient wards will help to increase patient satisfaction, a fundamental indicator in current health policies.

## Abbreviations

INAD: Institut de Neuropsiquiatria i Addiccions
PAR: Participatory Action Research
TR: Therapeutic Relationship
